# Supplementary material for: Chl1, an ATP-Dependent DNA Helicase, Inhibits DNA:RNA Hybrids Formation at DSB Sites to Maintain Genome Stability in S. pombe
Source: Int J Mol Sci. 2022 Jun 14;23(12):6631. doi: 10.3390/ijms23126631 (PMC9224301; doi:10.3390/ijms23126631)
Supplement: Supplementary file 1 [file ijms-23-06631-s001.zip › ijms-1673041-supplementary.pdf]

Supplementary materials

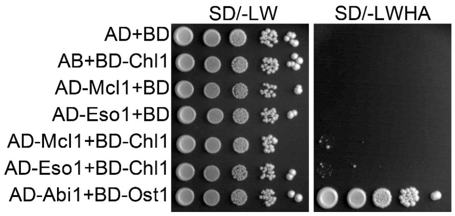

Figure S1. Chl1 does not interact with Mcl1 or Eso1. The interaction of Chl1 with Mcl1 or Eso1 was detected by Y2H. Tenfold serial dilutions of transformants were spotted on SD/-Leu-Trp media (control) and SD/-Leu-Trp-His-Ade media. The interaction between Abi1 and Ost1 is a contrast.

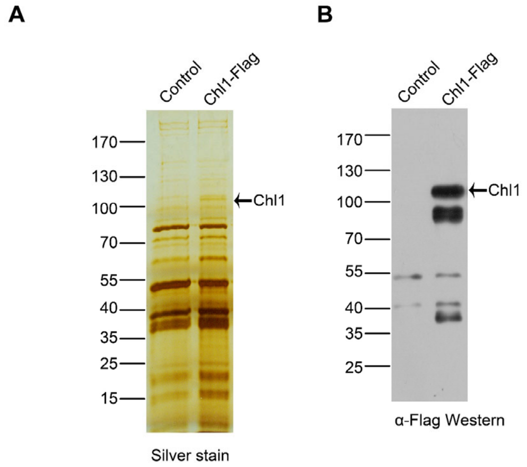

C

Bait: Chl1-Flag

Protein	Spectrum count	Percent coverage
Rpc37	4	17%
Chl1	270	73%

Figure S2. Characterization of the Chl1-Flag complex. (A) The purified Chl1-Flag complex was separated with 10% SDS PAGE gels for silver staining, an untagged strain served as contrast. (B) A portion of each sample from A was used for Western blot analysis with Flag antibody. (C) Affinity-purified Chl1-Flag complex and control material were used for Mass spectrometry analyses. The number of peptides and the percentage of Rpc37 and Chl1 that these peptides cover were presented.

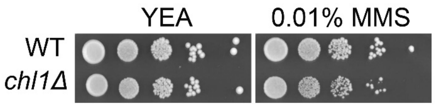

Figure S3. The *chl1Δ* strain is less sensitive to DNA damaging agent. Tenfold serial dilutions of strains were spotted on YEA medium containing the indicated concentration of DNA damaging agent.
